# Unmasking Acute Posterior Multifocal Placoid Pigment Epitheliopathy (APMPPE) Through Multimodal Imaging: A Case Report

**DOI:** 10.7759/cureus.86860

**Published:** 2025-06-27

**Authors:** Konstantinos Flindris, Chrysa Chatzipetrou, Eleni Papafotiou, Athanasios Kaliardas, Ioannis Koumpoulis, Ioannis Melissourgos

**Affiliations:** 1 Ophthalmology, General Hospital of Ioannina "G. Hatzikosta", Ioannina, GRC; 2 Medicine, Aristotle University of Thessaloniki, Thessaloniki, GRC

**Keywords:** acute posterior multifocal placoid pigment epitheliopathy, apmppe, multimodal imaging, non-invasive multimodal imaging, uveitis, white dot syndrome

## Abstract

Acute posterior multifocal placoid pigment epitheliopathy (APMPPE) is a rare, immune-mediated chorioretinal inflammatory condition typically affecting healthy young adults. It often presents with acute bilateral vision loss and multiple creamy placoid lesions at the level of the retinal pigment epithelium, frequently following a flu-like or viral prodrome. We report the case of a 24-year-old male who developed bilateral subacute vision loss, three days in duration, one month after a febrile upper respiratory infection. Fundus examination revealed multiple yellow-white placoid lesions in both eyes, and multimodal retinal imaging (optical coherence tomography (OCT), fundus autofluorescence (AF), and OCT angiography (OCTA)) confirmed features consistent with APMPPE. A comprehensive infectious and autoimmune workup was negative, and neuro-imaging showed no evidence of cerebral vasculitis. The patient was managed conservatively without immunosuppressive therapy. Over the course of two weeks, his visual acuity spontaneously improved from 20/65 (logarithm of the Minimum Angle of Resolution (logMAR) = 0.5) to 20/30 (logMAR = 0.2) in the right eye and from 20/25 (logMAR = 0.1) to 20/20 (logMAR = 0) in the left eye, with corresponding regression of the retinal lesions. This case highlights the importance of recognizing APMPPE in the appropriate clinical context through non-invasive multimodal imaging, distinguishing it from infectious or chronic placoid retinopathies, emphasizing the role of careful observation and follow-up in managing this condition with a favorable visual prognosis.

## Introduction

Acute posterior multifocal placoid pigment epitheliopathy (APMPPE) is an uncommon inflammatory chorioretinopathy first described by Gass in 1968 [1]. It is classified among the white dot syndromes, and there is primary inflammation of the choriocapillaris and secondary involvement of the retinal pigment epithelium (RPE) and photoreceptors [2]. This syndrome typically affects men and women equally in their second to fourth decades of life. APMPPE often presents as an abrupt, painless loss of vision in one or both eyes, accompanied by photopsias and scotomas, with funduscopic findings of multiple flat, creamy yellow-white placoid lesions at the level of the RPE and mid-sized vessels. These placoid lesions are usually concentrated in the posterior pole (within the vascular arcades), roughly one to two disc diameters in size [3]. In approximately 40-60% of cases, APMPPE follows a nonspecific viral prodrome, most commonly adenovirus, coxsackievirus, influenza, or Epstein-Barr virus, presenting as fever, upper respiratory symptoms, or malaise. The interval between systemic infection and the onset of ocular symptoms generally ranges from one to four weeks, with a mean latency of about two weeks, suggesting an immune-mediated pathogenesis triggered by molecular mimicry or immune complex deposition. APMPPE is also preceded by a vaccination in a significant proportion of cases. Although most cases are sporadic, APMPPE has been linked to certain HLA serotypes (notably HLA-B7 and HLA-DR2), indicating a possible genetic predisposition to this inflammatory response. The disease course is classically acute and monophasic, with new lesions sometimes appearing over a few weeks but typically no long-term recurrences. Most cases are self-limited, and visual symptoms tend to improve spontaneously over four to eight weeks as the placoid lesions heal, often leaving behind RPE pigmentary changes or atrophy [4]. However, APMPPE is clinically important not only because it can cause substantial transient visual impairment, but also due to its potential systemic associations. Notably, a subset of patients develop neurologic complications such as cerebral vasculitis, meningitis, or stroke, which can be sight- and life-threatening [5]. Multimodal retinal imaging is essential for confirming APMPPE and differentiating it from mimicking entities (e.g., infectious chorioretinitis like syphilitic placoid retinopathy). Given its rarity and the need to exclude mimicking conditions, reporting case experiences is valuable to refine our understanding and management of APMPPE. Herein, we present a case report of a young adult male with bilateral APMPPE following a viral prodrome, and we discuss the role of multimodal imaging in the diagnosis of APMPPE, the management approach, and the outcomes in the context of the current literature.

## Case presentation

A 24-year-old man with no significant past medical history (no prior surgeries, medications, or known systemic illnesses) presented to the emergency department with a chief complaint of blurry vision in both eyes for three days. The visual decline was subacute and painless. He denied any eye pain, redness, or flashes of light, but noted seeing “spots” in his central vision. One month prior to presentation, the patient had experienced a febrile illness with a maximum temperature of 39°C, accompanied by symptoms of an upper respiratory tract infection (sore throat, congestion, and myalgias) that resolved over about one week without any medication. He had no history of recent vaccinations, and there was no report of rash, arthralgia, or other systemic symptoms following that illness. There was no family history of ophthalmologic or autoimmune diseases.

**Figure 1:**
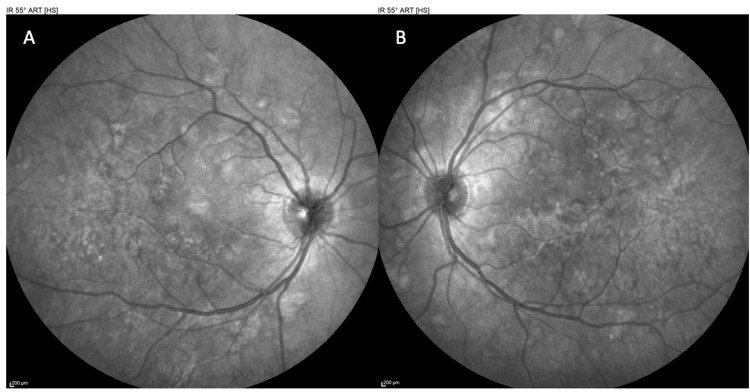
Infrared (IR) photography of the right eye (OD) (A) and left eye (OS) (B) at presentation, revealing multiple placoid retinal lesions scattered in the posterior pole

**Figure 2:**
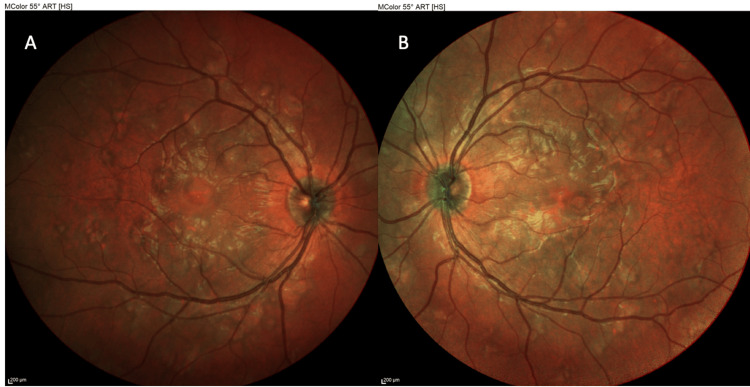
MultiColor fundus photography of the right eye (OD) (A) and left eye (OS) (B) at presentation, presenting multiple yellow-white, placoid retinal lesions scattered in the posterior pole

**Figure 3:**
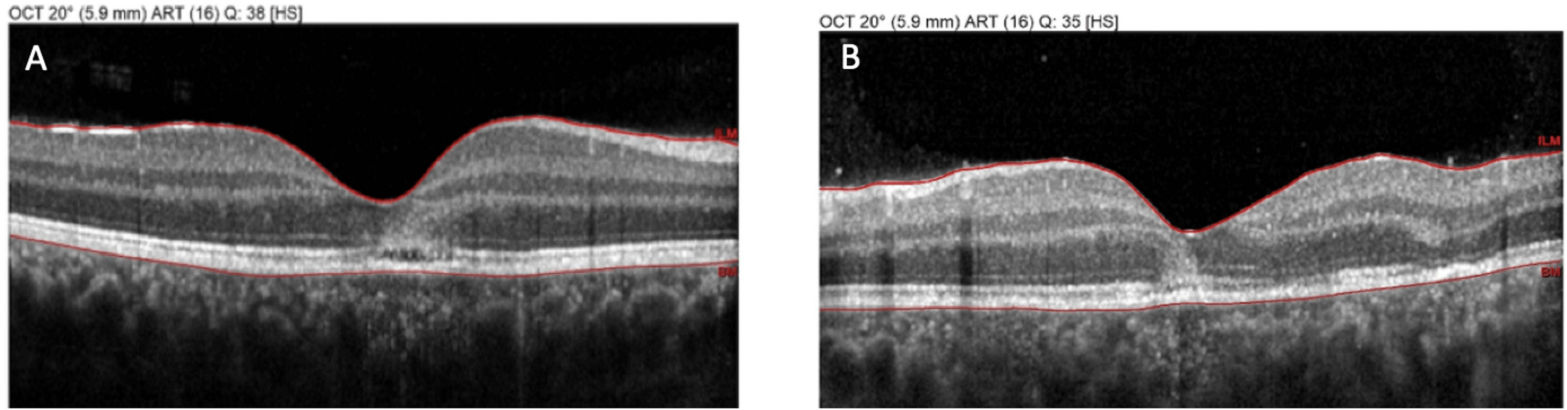
Optical coherence tomography (OCT) of the right eye (OD) (A) and left eye (OS) (B) at presentation, demonstrating disruption of the outer retinal layers and the angular sign of Henle fiber layer hyperreflectivity (ASHH) at the fovea, indicative of acute photoreceptor damage

On examination in the emergency department, his vital signs were normal, and systemic review was unremarkable aside from the visual symptoms. Best corrected visual acuity (BCVA) measured 20/65 (logarithm of the Minimum Angle of Resolution (logMAR) = 0.5) in the right eye (OD) and 20/25 (logMAR = 0.1) in the left eye (OS) in Snellen notation. Intraocular pressure was 11 mmHg in both eyes. Anterior segment examination of both eyes was unremarkable. Pupils were equal and reactive, with no relative afferent pupillary defect (RAPD) and full ocular motility. Dilated fundus examination of both eyes revealed multiple yellow-white, placoid retinal lesions scattered in the posterior pole. The lesions were approximately one optic disc diameter in size, flat, and had indistinct edges (Figures 1-2). The optic discs appeared slightly hyperemic but without swelling, and there was no retinal vasculitis or hemorrhage noted (Figure 3). The vitreous was clear with no inflammatory cells.

**Figure 4:**
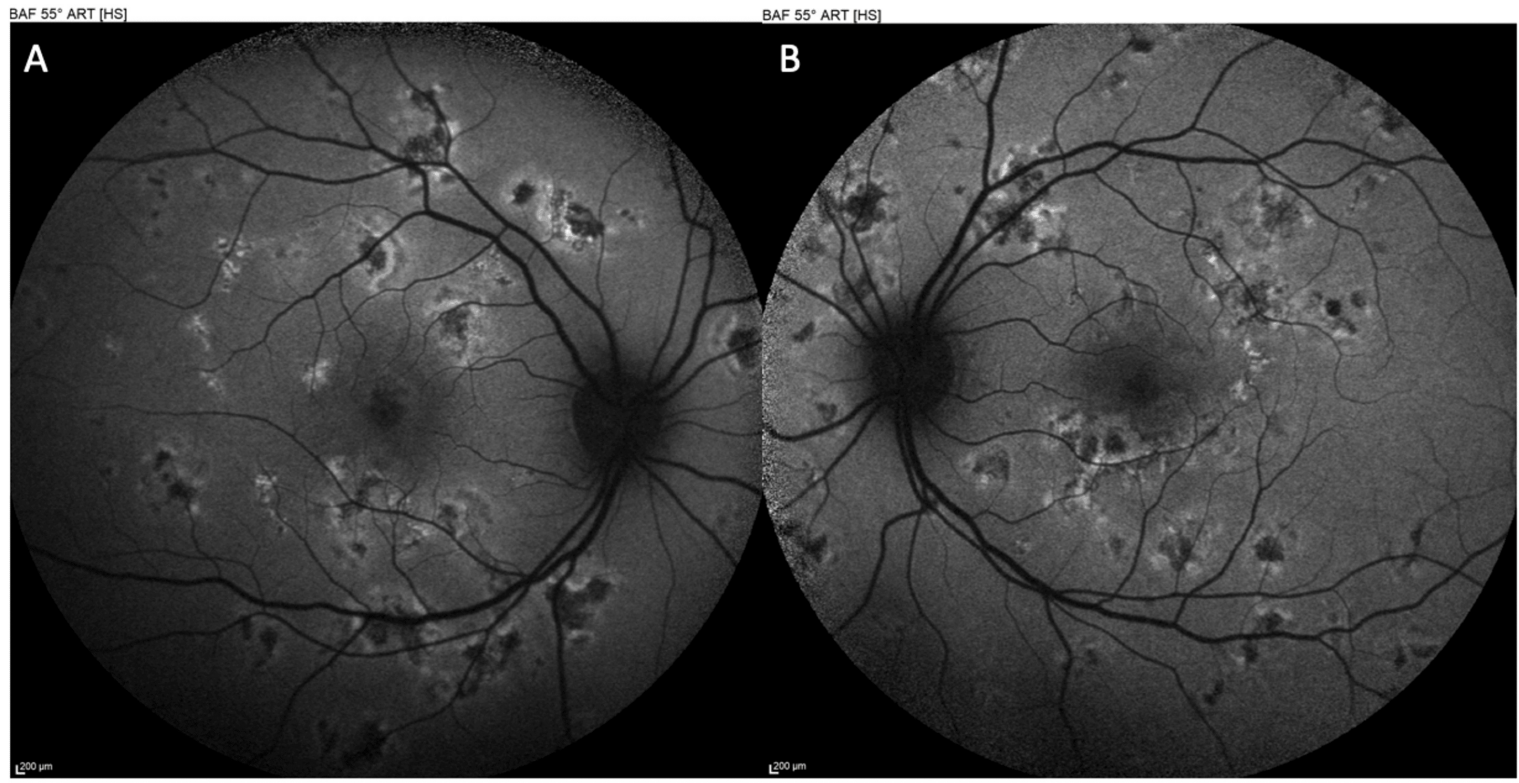
Autofluorescence (AF) of the right eye (OD) (A) and left eye (OS) (B) at presentation, showing hypoautofluorescent lesions, consistent with an acute disruption of the retinal pigment epithelium (RPE), with a faint hyperautofluorescent rim developing at their edges

**Figure 5:**
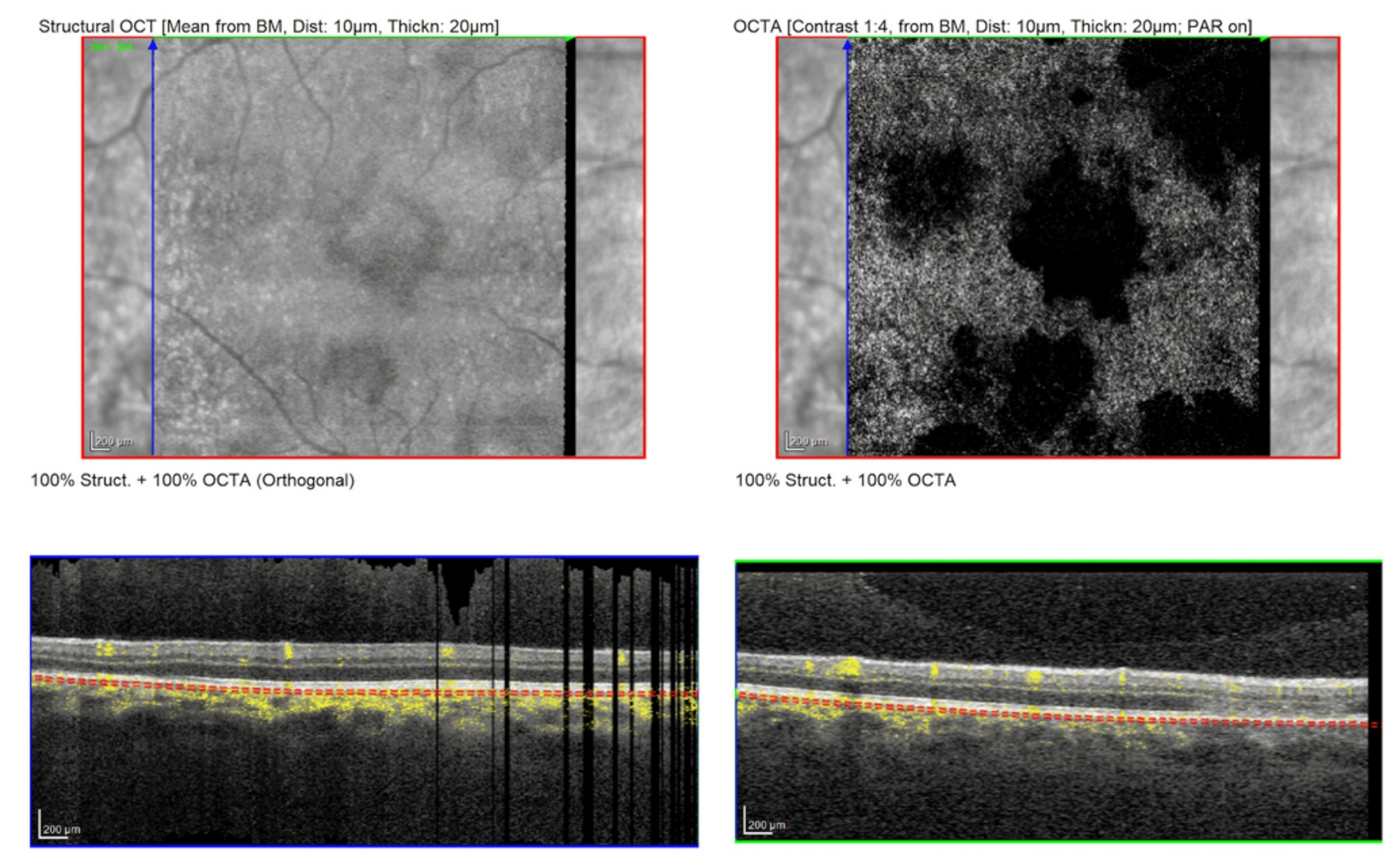
Optical coherence tomography angiography (OCTA) of the right eye (OD) at presentation, revealing multiple patchy areas of flow reduction (flow voids) at the level of the choriocapillaris corresponding to the placoid lesions

Non-invasive multimodal imaging was performed to further investigate the lesions. Optical coherence tomography (OCT) through the areas of active placoid lesions demonstrated disruption of the outer retinal layers. Specifically, the acute lesions corresponded to hyperreflective material at the level of the outer retina and RPE with attenuation of the ellipsoid zone (photoreceptor inner/outer segment junction) beneath the lesions. There was no significant subretinal fluid or retinal detachment noted on OCT. OCT at the OD and OS revealed the angular sign of Henle fiber layer hyperreflectivity (ASHH) at the fovea (Figure 3), which is a novel OCT biomarker indicative of acute photoreceptor disruption [6]. Fundus autofluorescence (AF) imaging showed that the active lesions were hypoautofluorescent relative to the surrounding retina, consistent with an acute masking effect on the RPE autofluorescence signal, while some lesions had a faint hyperautofluorescent rim developing at their edges (Figure 4). OCT angiography (OCTA) was also obtained, focusing on the choriocapillaris layer. OCTA revealed multiple patchy areas of flow reduction (flow voids) at the level of the choriocapillaris corresponding to the placoid lesions in both eyes (Figure 5). Notably, the regions of choriocapillaris flow deficit on OCTA were slightly larger in extent than the visible lesions on fundus exam or structural OCT, suggesting subclinical involvement of the choroidal circulation beyond the obvious lesions. These multimodal imaging findings (outer retinal disruption and ASHH on OCT, hypoautofluorescent placoid areas on AF, and choriocapillaris flow voids on OCTA) were all characteristic of acute APMPPE lesions. Based on the clinical presentation and imaging, a diagnosis of APMPPE was made.

A broad diagnostic workup was undertaken to exclude infectious or other inflammatory etiologies that can mimic APMPPE. Laboratory tests, including complete blood count, inflammatory markers, and specific infectious serologies, were all unremarkable (Table 1).

**Table 1:** Laboratory blood tests at presentation ACE, angiotensin-converting enzyme; pANCA, perinuclear anti-neutrophil cytoplasmic antibody; cANCA, cytoplasmic anti-neutrophil cytoplasmic antibody; RPR, rapid plasma reagin; RF, rheumatoid factor; TSH, thyroid-stimulating hormone

Laboratory Test	Value	Reference Range
White blood cells	6.82 × 10^3^ /μL	4-11 × 10^3^ /μL
Neutrophils	56%	40-75%
Lymphocytes	34.2%	20-45%
Monocytes	7.5%	2-10%
Eosinophils	1.9%	1-6%
Basophils	0.4%	0.2-1%
Red blood cells	5.15 × 10^6^ /μL	3.8-6 × 10^6^ /μL
Hemoglobin	14.2 g/dL	11.8-17.8 g/dL
Hematocrit	43.2%	36-52%
Mean corpuscular volume	83.9 fL	80-96 fL
Mean corpuscular hemoglobin	27.6 pg	26-32 pg
Mean corpuscular hemoglobin concentration	32.9 pg/dL	32-36 pg/dL
Platelets	244 × 10^3 ^/μL	140-450 × 10^3 ^/μL
INR	1	1-1.3
Activated partial thromboplastin time	30.2 seconds	26-36 seconds
Fasting blood sugar	93 mg/dL	70-115 mg/dL
HbA1c	5.5%	0-6%
Urea	28 mg/dL	0-50 mg/dL
Creatinine	0.9 md/dL	0.8-1.4 mg/dL
Potassium	4.5 mmol/dL	3.5-5.1 mmol/dL
Sodium	141 mmol/dL	136-146 mmol/dL
Magnesium	1.55 mEq/L	1.3-2.1 mEq/L
Calcium	9.7 mg/dL	8.2-10.5 mg/dL
Total proteins	6.7 g/dL	6.2-8.4 g/dL
Albumin	4.3 g/dL	3.5-5.1 g/dL
Total bilirubin	0.33 mg/dL	0.1-1.3 mg/dL
AST	32 IU/L	5-40 IU/L
ALT	43 IU/L	5-40 IU/L
γGT	23 IU/L	8-45 IU/L
ALP	118 IU/L	35-125 IU/L
LDH	213 IU/L	120-230 IU/L
CPK	115 IU/L	0-220 IU/L
CK-MB	15 IU/L	0-23 IU/L
Amylase	63 IU/L	28-100 IU/L
Uric acid	4.1 mg/dL	3.6-7.8 mg/dL
TSH	2.98 μIU/mL	0.35-4.94 μIU/mL
Free T4	1.00 ng/dL	0.70-1.48 ng/dL
Free T3	106.5 ng/dL	64-152 ng/dL
Total cholesterol	239 mg/dL	120-220 mg/dL
HDL-cholesterol	47 mg/dL	35-55 mg/dL
Triglycerides	96 mg/dL	30-160 mg/dL
Ferritin	156 ng/mL	0-300 ng/mL
B12	501 pg/mL	187-883 pg/mL
CRP	0.48 mg/dL	0-0.8 mg/dL
C3	123 mg/dL	79-152 mg/dL
C4	28.3 mg/dL	16-38 mg/dL
IgG	1088 mg/dL	751-1560 mg/dL
IgM	166.1 mg/dL	46-304 mg/dL
IgA	333 mg/dL	82-453 mg/dL
ACE	52 mg/dL	0-53 mg/dL
ANA	Negative	-
pANCA	Negative	-
cANCA	Negative	-
Anti-ds-DNA	Negative	-
Anti-cardiolipin, anti-beta2-glycoprotein, anti-GBM	Negative	-
RF	Negative	-
Anti-CCP	Negative	-
Anti HBV, HCV, HIV, HSV, VZV IgM, IgG	Negative	-
RPR	Negative	-
Anti-*Leishmania* IgM, IgG	Negative	-
Anti-*Toxoplasma* IgM, IgG	Negative	-
Anti-*Borrelia* IgM, IgG	Negative	-
QuantiFERON-TB Gold	Negative	-

Furthermore, a thorough neurological examination was completed and found to be normal (no focal deficits, no meningismus or encephalopathic signs). Magnetic resonance imaging (MRI) of the brain and orbits with gadolinium-contrast, as well as magnetic resonance angiography (MRA), were performed and showed no abnormalities - specifically, no signs of cerebral vasculitis, white matter lesions, or optic nerve enhancement. The absence of infectious findings, along with normal neuroimaging, supported the diagnosis of primary APMPPE and reduced concern for an alternative diagnosis such as infectious posterior uveitis or a neurological vasculitic process, and hence the need for specific treatment. Given the diagnosis of APMPPE and the fact that the patient’s central vision, although impaired, was still functional (20/65 in the worse eye), the decision was made to manage conservatively with close observation. No systemic corticosteroids or immunosuppressive therapy was initiated. The patient was counseled on the expected course of APMPPE - that it is typically self-limited and usually improves over a period of weeks - and was advised to report any new symptoms, especially any neurological complaints like headaches or focal deficits, immediately, and was scheduled for close follow-up.

**Figure 6:**
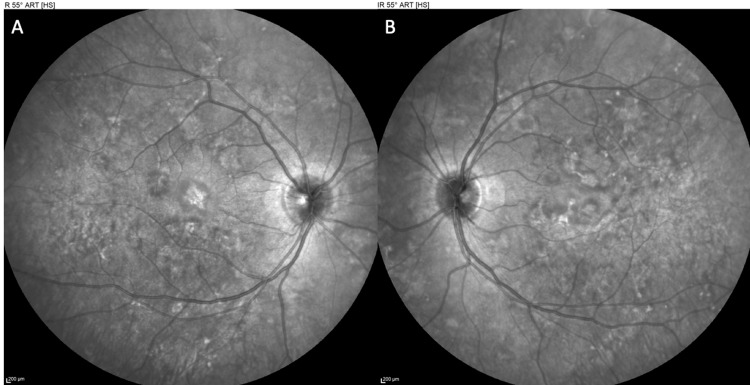
Infrared (IR) photography of the right eye (OD) (A) and left eye (OS) (B) at two-week follow-up, suggesting fading of the retinal lesions

**Figure 7:**
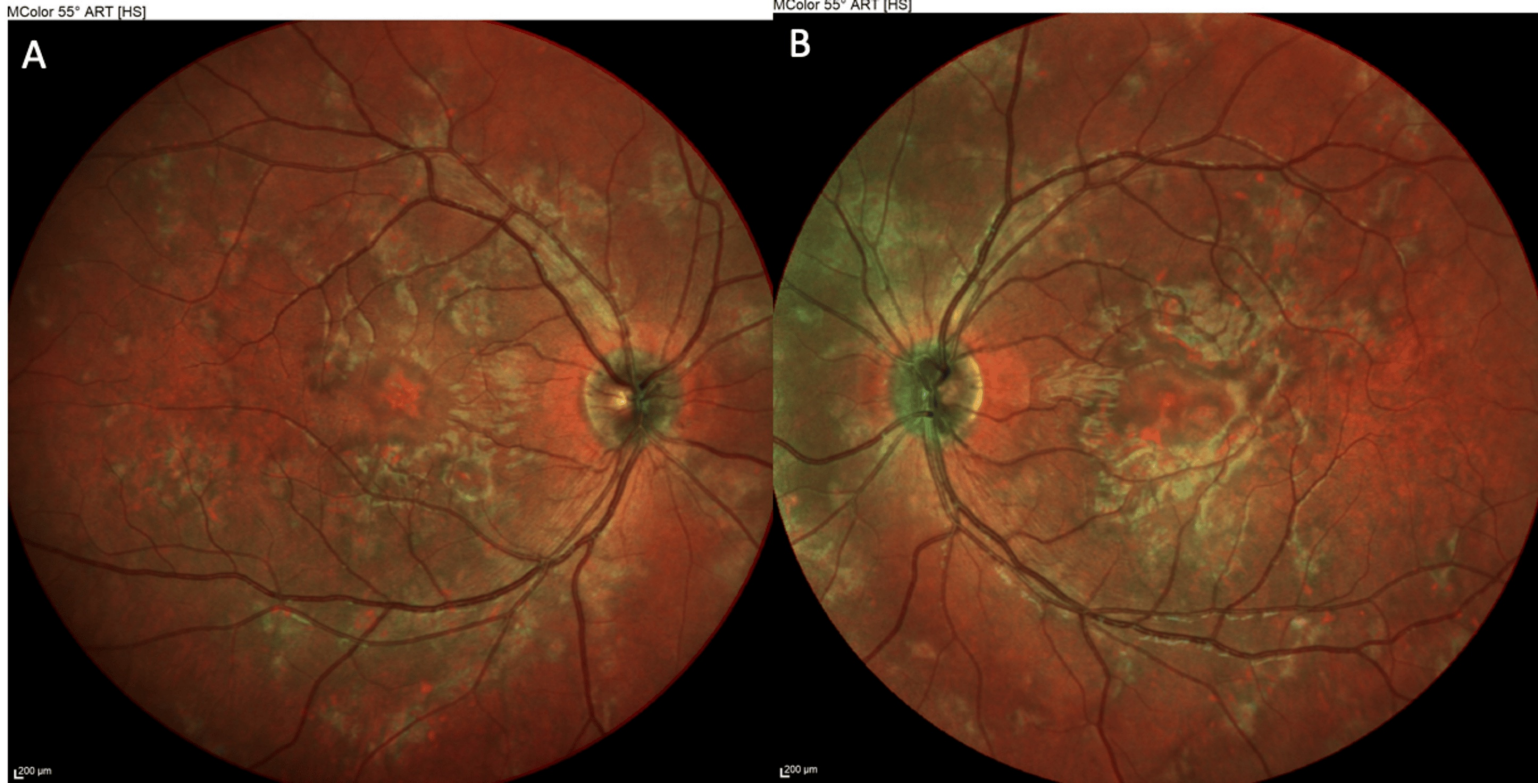
MultiColor fundus photography of the right eye (OD) (A) and left eye (OS) (B) at two-week follow-up, indicating fading of the retinal lesions

Over the ensuing two weeks, the patient noted steady improvement in his vision. At the two-week follow-up visit, his BCVA had improved to 20/30 (logMAR = 0.2) OD and 20/20 (logMAR = 0) OS. He reported resolution of the central scotomas and only mild residual blurriness in the right eye. Repeat fundus examination showed that most of the placoid lesions had faded. Instead of the distinct yellow-white plaques seen earlier, there were irregular areas of mild hyperpigmentation and RPE mottling in those locations, indicating healing lesions and no new lesions had appeared (Figures 6-7). The optic disc hyperemia had resolved, and there was still no sign of any retinal vasculitis or vitreous inflammation.

**Figure 8:**
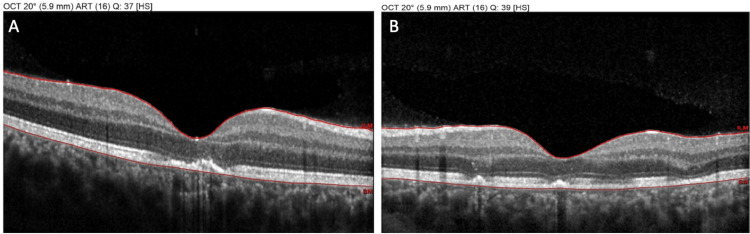
Optical coherence tomography (OCT) of the right eye (OD) (A) and left eye (OS) (B) at two-week follow-up, presenting partial restoration of the ellipsoid zone and of the ASHH, and some focal defects remaining that correspond to areas of retinal pigment epithelium (RPE) atrophy

**Figure 9:**
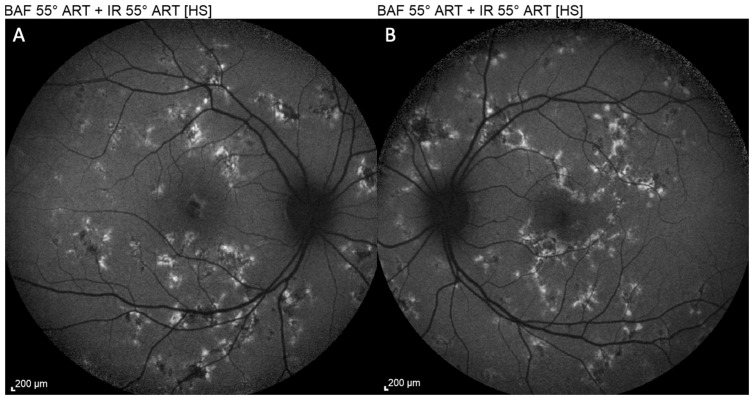
Autofluorescence (AF) of the right eye (OD) (A) and left eye (OS) (B) at two-week follow-up, demonstrating regression of the retinal lesions and of the retinal pigment epithelium (RPE) disruption

**Figure 10:**
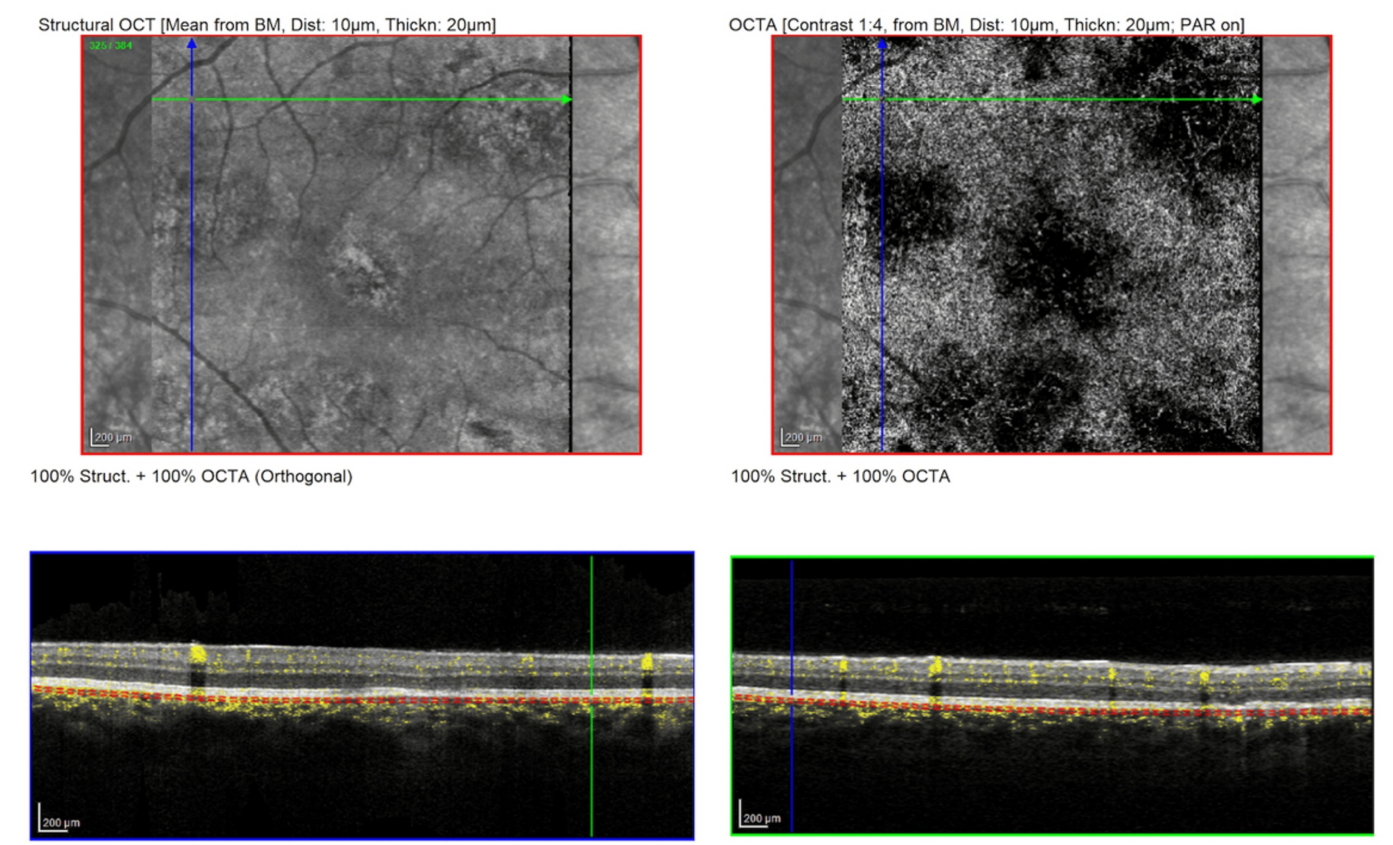
Optical coherence tomography angiography (OCTA) of the right eye (OD) at two-week follow-up, revealing significant resolution of the choriocapillaris flow voids, with restoration of flow in areas that were previously ischemic, correlating with the patient’s visual recovery

Follow-up OCT imaging demonstrated reconstitution of the outer retinal architecture in areas that previously showed photoreceptor disruption: the hyperreflective material in the outer retina had cleared, and the ellipsoid zone band showed partial restoration, though some focal defects remained that correspond to areas of RPE atrophy (Figure 8). Choroidal thickness, which was slightly elevated during the acute phase, appeared to have normalized on enhanced-depth OCT scans. Fundus AF at follow-up showed a mixed pattern: many lesions that were previously hypoautofluorescent had become isoautofluorescent or even mildly hyperautofluorescent, reflecting RPE metabolic activity during recovery, and a few persistent hypoautofluorescent spots corresponding to residual RPE damage (Figure 9). Follow-up OCTA confirmed that the choriocapillaris flow voids had largely resolved, with partial restoration of flow in areas that were previously ischemic, correlating with the patient’s visual recovery (Figure 10). These findings were all consistent with the expected evolution of APMPPE lesions in the healing phase.

The patient’s clinical course was benign. No treatment was required, and his condition improved spontaneously. He continued to be monitored for several months. By three months after presentation, his right eye visual acuity had further improved to 20/25 (logMAR = 0.1) OD and remained 20/20 logMAR = 0) OS. Mild residual RPE pigmentary changes were present in the right eye macula, but there was no development of choroidal neovascularization or other complications. The patient did not experience any neurologic symptoms at any point. This favorable outcome was consistent with the generally good prognosis of APMPPE in immunocompetent patients without CNS involvement.

## Discussion

This case illustrates a classic presentation of APMPPE and underscores several important aspects regarding the role of non-invasive multimodal imaging in the diagnosis and the conservative management. APMPPE is believed to be an immune-mediated chorioretinal inflammatory process often triggered by a preceding infection. In our patient, the history of a high fever and upper respiratory tract infection one month prior is a plausible precipitating event. Similar temporal associations between a flu-like illness or vaccinations and subsequent APMPPE have been well documented [7-10]. The leading hypothesis is that a viral or other antigenic stimulus incites an immune response that cross-reacts with the choroidal vasculature, particularly the choriocapillaris, causing an acute choroidal ischemia and secondary RPE damage [11]. Histopathologic studies and imaging evidence support this mechanism: the hallmark of APMPPE is primary ischemia of the choriocapillaris (inner choroid), resulting in the overlying RPE and photoreceptor disruption [12]. On OCTA, our patient demonstrated areas of choriocapillaris flow void corresponding to the lesions, which is consistent with reported OCTA findings in APMPPE. As the inflammation resolves and choroidal perfusion improves, the outer retina often recovers, explaining the spontaneous visual improvement seen in most cases. APMPPE has also been associated with certain HLA serotypes (e.g., HLA-B7, DR2) and occasionally with systemic inflammatory conditions, further indicating an immune-genetic predisposition [13].

A critical aspect in managing this case was establishing the correct diagnosis and excluding other entities that can present with placoid retinal lesions. The differential diagnosis for multiple placoid white lesions in the fundus is broad and includes both non-infectious white dot syndromes and infectious posterior uveitides [14]. Non-invasive multimodal imaging was invaluable in our patient’s diagnostic confirmation and in monitoring the disease course. In our case, we relied on non-invasive OCTA to assess choroidal circulation, which confirmed reversible flow deficits at the level of the choriocapillaris matching the lesions. The OCT findings in this case (outer retinal hyperreflectivity with ellipsoid zone disruption in acute lesions and ASHH) are characteristic of APMPPE, as documented in prior case series. As lesions heal, OCT typically shows either restoration of the outer retinal layers or residual thinning and RPE bumpiness in areas where damage was irreversible. We observed both patterns in our patient: most areas showed near-complete structural recovery on OCT, correlating with visual improvement, while a few spots (particularly in the right eye) demonstrated persistent RPE irregularity corresponding to slight scotomas. Fundus AF imaging also corroborated the stage of disease: initially, the lesions were hypoautofluorescent (suggesting blocked or absent RPE autofluorescence due to acute lesion material), and later, a rim of hyperautofluorescence developed around some lesions, a finding attributed to RPE metabolic stress and lipofuscin accumulation at the healing edges. Eventually, areas of resolved lesions can become uniformly hypoautofluorescent if permanent RPE atrophy ensues. These multimodal imaging signatures help differentiate APMPPE from other conditions. Thus, imaging not only supported our diagnosis but also provided objective measures of recovery.

An essential consideration in this case was the management strategy. Currently, there is no consensus on the optimal treatment for APMPPE. Because APMPPE is typically self-limited, many cases have been managed with observation alone, and spontaneous recovery of vision within four to eight weeks is common [15]. Our case adds to the body of evidence that a conservative approach can be successful in uncomplicated APMPPE. The patient’s vision improved from 20/65 (logMAR = 0.5) to 20/30 (logMAR = 0.2) in the worse eye within two weeks without any intervention. Some clinicians advocate for corticosteroid therapy (systemic steroids) in APMPPE, particularly in cases with macular-threatening lesions or severe vision loss, with the aim of reducing inflammation and hastening visual recovery. Indeed, there are reports of using high-dose oral prednisone or even intravenous methylprednisolone in APMPPE, and some patients appear to recover faster with treatment. However, controlled trials are lacking given the rarity of the disease. Additionally, neurologic involvement is a game-changer in APMPPE management - if patients have symptoms like headaches or any evidence of cerebral vasculitis (e.g., on MRI or lumbar puncture), most experts will treat aggressively with systemic corticosteroids and even immunosuppressants, given the risk of stroke [16]. Consequently, our patient was monitored closely for any such signs, and since he remained neurologically intact and was improving, observation was maintained. Another reason to avoid unnecessary steroids is that, unlike chronic placoid disorders (serpiginous, relentless placoid), APMPPE usually does not require long-term immunosuppression - it rarely recurs once resolved. Prolonged steroid use carries significant side effects, so one must balance the potential benefits.

## Conclusions

In conclusion, we report a case of bilateral APMPPE in a young male following a febrile viral illness, which was managed successfully with conservative measures and resulted in near-complete visual recovery. This case underscores several key points. First, a thorough evaluation is critical for any placoid retinopathy: infectious etiologies such as syphilis and tuberculosis must be excluded, and careful systemic and neurologic assessment must be conducted to rule out associated cerebral vasculitis. Second, non-invasive multimodal retinal imaging (OCT, AF, OCTA) is extremely helpful in confirming the diagnosis and tracking the resolution of lesions in APMPPE. The characteristic imaging features - outer retinal disruption and ASHH on OCT with corresponding hypoautofluorescent placoid lesions and choriocapillaris flow deficits - can clinch the diagnosis and distinguish APMPPE from its mimics. Third, most APMPPE cases have a favorable prognosis with supportive care; in our patient, significant visual improvement occurred within weeks without steroid therapy. Treatment should be individualized based on lesion location, severity, and systemic involvement. High-dose corticosteroids are reserved for severe cases, especially those with macular involvement or neurologic complications, while milder cases can be closely observed given the self-limited nature of the disease. Lastly, this case highlights the importance of prompt recognition of APMPPE by emergency and eye care providers. Early diagnosis allows for appropriate counseling of the patient regarding the benign course of the condition, avoids unnecessary interventions, and ensures vigilant monitoring for the rare but serious complications. Through this case report, we aim to contribute to better recognition and understanding of APMPPE, aiding clinicians who encounter similar presentations in the future.
